# Increased power generation in supercapacitive microbial fuel cell stack using Fe—N—C cathode catalyst

**DOI:** 10.1016/j.jpowsour.2018.11.069

**Published:** 2019-02-01

**Authors:** Carlo Santoro, Mounika Kodali, Najeeb Shamoon, Alexey Serov, Francesca Soavi, Irene Merino-Jimenez, Iwona Gajda, John Greenman, Ioannis Ieropoulos, Plamen Atanassov

**Affiliations:** a Department of Chemical and Biological Engineering, Center for Micro-Engineered Materials (CMEM), University of New Mexico, Albuquerque, NM, 87131, USA; b Department of Chemistry “Giacomo Ciamician”, Alma Mater Studiorum – Università, di Bologna, Via Selmi, 2, 40126, Bologna, Italy; c Bristol BioEnergy Centre, Bristol Robotics Laboratory, T-Block, UWE, Coldharbour Lane, Bristol, BS16 1QY, UK; d Biological, Biomedical and Analytical Sciences, UWE, Coldharbour Lane, Bristol, BS16 1QY, UK

**Keywords:** Supercapacitor, Microbial fuel cell, Galvanostatic discharges, Fe-based catalyst, Long terms stability

## Abstract

The anode and cathode electrodes of a microbial fuel cell (MFC) stack, composed of 28 single MFCs, were used as the negative and positive electrodes, respectively of an internal self-charged supercapacitor. Particularly, carbon veil was used as the negative electrode and activated carbon with a Fe-based catalyst as the positive electrode. The red-ox reactions on the anode and cathode, self-charged these electrodes creating an internal electrochemical double layer capacitor. Galvanostatic discharges were performed at different current and time pulses. Supercapacitive-MFC (SC-MFC) was also tested at four different solution conductivities. SC-MFC had an equivalent series resistance (ESR) decreasing from 6.00 Ω to 3.42 Ω in four solutions with conductivity between 2.5 mScm^−1^ and 40 mScm^−1^. The ohmic resistance of the positive electrode corresponded to 75–80% of the overall ESR. The highest performance was achieved with a solution conductivity of 40 mS cm^−1^ and this was due to the positive electrode potential enhancement for the utilization of Fe-based catalysts. Maximum power was 36.9 mW (36.9 W m^−3^) that decreased with increasing pulse time. SC-MFC was subjected to 4520 cycles (8 days) with a pulse time of 5 s (i_pulse_ 55 mA) and a self-recharging time of 150 s showing robust reproducibility.

## Highlights

• Supercapacitive features of 1 L microbial fuel cell were investigated.
• Iron based catalyst was used at the cathode to increase the potential.
• The positive electrode shown high ESR and high capacitance.
• Maximum power output from the supercapacitive MFC was 36.9 mW (36.9 W m^−3^).
• 4520 cycles of discharge/self-recharge were presented showing system stability.

## 1 Introduction

Microbial fuel cells (MFC) use bacteria to produce electric power by simultaneously degrading organic compounds and thereby treating wastewater [[1], [2], [3]]. This is an important aspect for this technology to be particularly useful for wastewater treatment applications with beneficial environmental impact [[1], [2], [3]]. Despite the numerous reports on significant improvements in microbial fuel cells, in terms of power output and water treatment, several issues still need to be solved before large-scale commercialization [4].

Considering the anode electrode, materials need to satisfy several physical, chemical and economical characteristics in order to be suitable [[5], [6], [7]]. Durability in long-term operations, biocompatibility, low cost, high electrical conductivity, resistance to corrosion, high surface area to enhance the bacteria/electrode interface and hydrophillicity to accommodate bacteria, are some of the main features of the anode material other than also being environmentally friendly [[5], [6], [7]]. Anodes used are generally carbonaceous-based [[8], [9], [10], [11]] or stainless steel-based [[12], [13], [14]] despite successful experiments have been carried out using other metal surfaces (e.g. copper [15], silver [15], titanium [16], etc). The kinetics of the oxidation processes occurring at the anode electrode are still low, especially when real wastewater is used, mainly due to the complexity of the substrate used [17]. Moreover, bacterial electron transfer is a topic that is still highly disputed within the international community [18,19].

In parallel, the cathode materials and the reduction reaction occurring on the electrode have also seen several improvements over the years, even though several limitations remain unsolved [20,21]. As oxidant, several alternatives have been proposed but oxygen is the most common due to the high reduction potential, natural availability in atmosphere at high concentration therefore not contributing to an extra weight or cost for the system operations [22]. The oxygen reduction reaction (ORR) follows two different pathways in function of the electrolyte, acidic or alkaline [23,24]. In both pathways, the reaction can involve 2 e^−^, 2 × 2 e^−^ and direct 4 e^−^ transfer mechanism [23,24]. In acidic environments, the 2 e^−^ mechanism transforms O_2_ into H_2_O_2_ that is chemically or electrochemically converted into H_2_O (2 × 2 e^−^). A direct 4 e^−^ transfer can occur with conversion of O_2_ directly in H_2_O. In alkaline environment, the 2 e^−^ mechanism transforms O_2_ into HO_2_^−^ that is chemically or electrochemically converted into OH^−^ (2 × 2 e^−^). A direct 4 e−transfer can occur with conversion of O_2_ directly in OH^−^. H^+^ is a reagent in acidic media while OH^−^ is a reagent in alkaline media and therefore extreme pH environments are preferred during the ORR. Bioelectrochemical systems need to operate in circumneutral pH level environments to preserve bacterial activity and therefore the ORR in neutral media is particularly hindered with extremely slow kinetics [[23], [24], [25]].

Catalysts are therefore used to accelerate the reaction at the cathode. Similarly to the anode materials, cathode catalysts need to be low cost and durable in polluted environments [[26], [27], [28], [29]]. Biotic catalysts such as enzymes and bacteria have been implemented with success [25,[30], [31], [32], [33], [34]] but enzymes are not durable in polluted environments and bacterial kinetics is not as high as the one obtained by the abiotic catalysts [35]. Abiotic catalysts are more utilized and can be classified in three main groups depending from the presence of metal and/or the presence of platinum group metal (PGM): i) carbonaceous materials; ii) platinum group metal (PGM) catalysts; iii) platinum group metal-free (PGM-free) catalysts [[27], [28], [29]]. Carbonaceous materials need to be highly conductive and have high surface area in order to accelerate the ORR. Different carbonaceous materials such as carbon black [[36], [37], [38]], carbon aerogel [39], carbon nanofibers [40], activated carbon [[41], [42], [43], [44]], graphene [45,46] and carbon nanotubes [47,48] have been used as cathode catalyst or cathode support. Activated carbon seems to be the more suitable for large-scale applications due to commercial availability, low cost and an effective electrochemical activity towards ORR [[41], [42], [43], [44]]. PGM catalysts were extensively used during the beginning of the development of the bioelectrochemical technologies because of the existence of the electrodes used in the more mature abiotic fuel cells fed with hydrogen and methanol [26,49,50]. Now, these materials are practically abandoned because of the exorbitant cost, low natural abundance and high affinity with anions that leads to fast poisoning and deactivation of the catalyst [[51], [52], [53]]. More recently, PGM-free materials based on the utilization of earth abundant transition metals such as Fe, Co, Mn and Ni have been exploited showing very high electrochemical performance in neutral media, high durability and low affinity with anions and pollutants and also low cost [[54], [55], [56], [57], [58], [59], [60], [61], [62], [63], [64]]. It was shown using rotating ring disk electrode (RRDE) technique and air-breathing cathode MFC that Fe-based catalysts were the most performing compared to Co, Mn and Ni with Co being the second best [65,66]. PGM-free are the best performing catalysts ever utilized in the MFC cathode and therefore remain the best choice for scientists in order to have the higher power output from MFCs [[27], [28], [29]].

Another challenge related with MFCs is the low power generated by the system that should be harvested in order to be utilized for practical applications. Therefore, although successful examples of practical applications were shown and published in the literature [[67], [68], [69], [70], [71]], several improvements still need to be done. As the power generated is low, usually MFCs are connected with external energy harvesters that are able to boost voltage and current in order to power applications [[67], [68], [69], [70], [71]]. Recently, it was shown that utilizing the anode and cathode electrodes of the MFC as the negative and positive electrodes of an internal supercapacitor [[72], [73], [74]] in order to boost the power performance, is possible. Supercapacitive anodes were also studied in other experiments reported in the literature [[75], [76], [77]]. The supercapacitive microbial fuel cell (SC-MFC) was able to generate high current pulse discharges and high power generation. Recently, the anodes and cathodes electrodes of a 1-L scale MFC stack with ceramic separators were used as electrodes of a self-charged supercapacitor operating in aqueous media [78]. This is a novel and interesting area that requires further improvement in electrode materials and system performance. In order to implement the SC-MFC into practical applications, the effect of the supercapacitive mode needs to be studied in long-term operations to check the durability of the system.

An SC-MFC ceramic stack was equipped with cathodes having iron-based catalysts in order to enhance the power output. The SC-MFC stack is here tested in supercapacitive mode at different electrolyte solution conductivities with ohmic drops and apparent capacitive features identified during the galvanostatic discharges. Discharges at different pulse time were done and analyzed. Power curves are also presented. For the first time, durability tests with 4520 cycles (9 days) of discharge and self-recharge are reported and discussed.

## 2 Materials and method

### 2.1 Electrode composition

Each SC-MFC consisted of an anode, a cathode and a ceramic membrane/separator. In the supercapacitive mode, the anode acts as negative electrode and the cathode as positive electrode of an internal supercapacitor. A photo as well as a drawing of the SC-MFC employed herein, can be found in Ref. [78]. The membrane used to separate the anode and cathode was a cylindrical ceramic separator with a height of ≈4 cm, an internal diameter of ≈2 cm and an external diameter of ≈2.3 cm and therefore the average thickness of the ceramic was ≈0.3 cm. The anode was fabricated using carbon veil (30 g m^−2^) as raw material with geometric area of 240 cm^−2^. The carbon veil was folded and wrapped on the external surface of each ceramic cylinder. Titanium wire was used to wrap the carbon veil and as final current collector. The cathode instead was inserted on the internal surface of the cylindrical ceramic.

Iron-based material was used as cathode catalyst for enhancing the ORR. Cathodic Fe—N—C catalyst was synthesized by modified Sacrificial Support Method (SSM) [[79], [80], [81]]. Initially a dispersion of two nitrogen-containing organic precursors: Nicarbazin (NCB) and Aminoantipirine (AAPyr) in water was deposited on the surface of high surface area silica (CabOSil, LM150 ∼ 150 m^2^ g^−1^). The obtained suspension of silica and organic precursors was mixed together by low energy ultrasonic treatment. The calculated amount of Fe(NO_3_)_3_*9H_2_O was added to homogeneous suspension in order to achieve a mass ratio between of iron nitrate and organics as 1:8. The water was evaporated at T = 65 °C under permanent ultrasonic treatment. Dry composite mixture was ground with agate mortar and pestle. Fine powder was heat treated in nitrogen atmosphere (UHP, flow rate of 100 ccm) at T = 915 °C for 45 min. Silica was removed with 25 wt % of HF for ∼20 h. Catalyst was washed by DI water until neutral pH and dried at T = 85 °C for ∼12 h. Obtained powder was additionally heat-treated in NH_3_ atmosphere (10% NH_3_, flow rate of 100ccm) at T = 955 °C for 30 min. The surface morphology and surface chemistry of the iron catalysts based on NCB and AAPyr was previously described in detail in Refs. [56,81], respectively.

The cathode was in air-breathing configuration and built as previously described [[56], [57], [58], [59], [60]]. Particularly, the cathode was prepared mixing activated carbon (AC, Norit SX Ultra, Sigma Aldrich), carbon black (CB, Alfa Aesar) and polytetrafluorethylene (PTFE, 60 wt% solution, Sigma Aldrich) all together using a blender. AC, CB and PTFE were mixed in percentage weight of 70%, 10% and 20% from previous cathode optimizations [[56], [57], [58], [59], [60]]. Each of the three ingredients has a particular role within the cathode matrix. In fact, AC was found to have relatively high catalytic activity towards ORR in neutral media and to be a promising, low cost and durable carbon support for the cathode matrix [[56], [57], [58], [59], [60]]. CB was shown to be important for the matrix increasing the overall conductivity of the cathode [37]. At last, PTFE is the most widely used binding agent for fabricating cathode in MFCs due to its low cost, moreover PTFE has hydrophobic property that is beneficial for promoting the three phase interface (TPI) within the electrode thus enhancing the presence of oxygen in gas phase [[56], [57], [58], [59], [60]]. AC/CB/PTFE were then mixed with iron-based catalyst and then pressed on a stainless-steel mesh used as cathode current collector. The final loading on the cathode was 41 mg cm^−2^ consisting of 1 mg cm^−2^ of iron-based catalyst and 40 mg cm^−2^ of AC/CB/PTFE.

### 2.2 Supercapacitive microbial fuel cell fabrication and operating conditions

The SC-MFC stack was contained into a plastic box with an empty volume 1 L (operating volume). This plastic box contained 28 MFCs that were electrically connected in parallel. The image of the used stack was previously presented [78]. The decision of connecting them in parallel was dictated by the fact that all the MFCs were sharing the same electrolyte. The SC-MFC stack was inoculated using activated sludge obtained from the Albuquerque SouthEast Reclamation facility in Albuquerque (NM, USA). The MFC stack was connected to an external resistance of 33 Ω. Few additions of sodium acetate were done over time till the MFC stack had a stable voltage output. The MFC stack was fed in continuous flow with a reservoir of activated sludge, potassium phosphate buffer and sodium acetate within a 4 L tank and an average flow of 20 mL min^−1^ using the peristaltic pump (MasterFlex 7523, ColePalmer). The MFC stack was tested with four solution conductivities measuring 2.5 mS cm^−1^, 13 mS cm^−1^, 22 mS cm^−1^ and 40 mS cm^−1^. The lower solution conductivity tested (2.5 mS cm^−1^) referred to the utilization of a solution containing only activated sludge. After testing the supercapacitive-MFC with electrolyte having 2.5 mS cm^−1^ as ionic strength, the solution conductivities were increased gradually by adding phosphate buffer solution and sodium acetate. After changing the solution, the MFC stack was put under a constant external resistance (33 Ω) for the necessary time to reach new stable voltage output. The SC-MFC stack run at room temperature (22 ± 2 °C) over the entire operations.

### 2.3 Electrochemical measurements

Tests were performed in supercapacitive mode. In this case, the electrodes were considered as the negative and positive electrode of an internal self-charged supercapacitor as previously shown [78]. After the voltage output of the SC-MFC stack was stable at each solution conductivity investigated, the SC-MFC stack was left in open circuit voltage (OCV) with no resistance for at least a period of time of 12 h till the OCV reached stability (±1 mV) before performing galvanostatic (GLV) discharges. The instrument used for the GLV discharges was an SP-50 Biologic Potentiostat. The electrodes were connected as follows: parallel connected cathodes to the working channel, parallel connected anodes to counter channel and Ag/AgCl 3 M KCl placed in the middle of the box to the reference channel. After voltage stabilization, discharges were performed for a determined time (**t**_**pulse**_) at certain applied current (**i**_**pulse**_). The **t**_**pulse**_ utilized were 5 s, 2 s, 1 s, 0.2 s.

Different parameters were measured during the GLV discharges. The initial voltage in which the SC-MFC stack is before GLV discharges, is named as **V**_**max,OC**_. Instantaneous and vertical change in voltage caused by ohmic resistance (**ΔV**_**ohmic,stack**_) of electrodes and the electrolytes was recorded when the GLV discharge occur. A new value, named **V**_**max**_ is therefore reached.

The maximum voltage or **V**_**max**_ was measured using equation (1).

(1) $$V_{max}=\;V_{max,OC}-\;\Delta V_{ohmic,stack}$$

The ohmic resistances were measured through the equivalent series resistance (**ESR**) calculated using equation (2).

(2) $$ESR=\;\frac{\Delta V_{ohmic,stack}}{i_{pulse}}$$

The resistances of the negative and positive electrode of the internal supercapacitor were calculated through equation (3) and equation (4).

(3) $$R_{NE}=\;\frac{\Delta V_{ohmic,NE}}{i_{pulse}}$$

(4) $$R_{PE}=\;\frac{\Delta V_{ohmic,PE}}{i_{pulse}}+R_{U}$$

The possibility of measuring **R**_**NE**_ and **R**_**PE**_ separately was due to the utilization of the reference electrode inserted within the electrolyte solution. **R**_**PE**_ also takes into account the contribution of an uncompensated resistance (**R**_**U**_) due to the utilization of a ceramic separator between the cathode and the electrolyte.

After the ohmic drop (**ΔV**_**ohmic,stack**_), the stack voltage decreased over time due to the capacitive behavior of the box (**ΔV**_**capacitive,stack**_). The stack apparent capacitance (**C**_**stack**_) was calculated using equation (5) where **s** is the variation of voltage over time.

(5) $$C_{stack}=\;\frac{i_{pulse}}{s}=\;\frac{i_{pulse}}{\frac{dV}{dt}}$$

Once again, as the anode and cathode discharge profiles were monitored separately, the apparent capacitance of the negative electrode (**C**_**NE**_) and the positive electrode (**C**_**PE**_) can be calculated separately as shown in equation (6) and equation (7).

(6) $$C_{NE}=\;\frac{i_{pulse}}{\frac{dV_{NE}}{dt}}$$

(7) $$C_{PE}=\;\frac{i_{pulse}}{\frac{dV_{PE}}{dt}}$$

Stack apparent capacitance (**C**_**stack**_) and separate electrode apparent capacitance (**C**_**NE**_ and **C**_**PE**_) are related through the following equation (8). We used the term “apparent” capacitance because the voltage (or potential) variation over the discharges cannot be due only to “pure” electrostatic discharge of the double layer. Faradaic reactions related to the MFC operations can also contribute, especially at the lowest currents.

(8) $$C_{stack}={\left(\frac{1}{C_{NE}}+\frac{1}{C_{PE}}\right)}^{-1}$$

Maximum power for the SC-MFC was calculated using equation (9):

(9) $$P_{max}=\;V_{max}\;\times \;i_{pulse}\;\;$$

**P**_**max**_ considers the maximum power that can be attained neglecting the capacitive losses in the SC-MFC. The power of a specific pulse (**P**_**pulse**_) for the duration of **t**_**pulse**_ is then lower than **P**_**max**_ because the capacitive losses that take place during the discharges are considered. **P**_**pulse**_ can be calculated equation (10) and it is the ratio between energy during the pulse (**E**_**pulse**_) and the time (**t**_**pulse**_).

(10) $$P_{pulse}=\;\frac{i_{pulse}\;\int_{0}^{t}Vdt}{t_{pulse}}=\;\frac{E_{pulse}}{t_{pulse}}$$

**P**_**max**_ and **P**_**pulse**_ were normalized to the box volume.

## 3 Results and discussion

### 3.1 Analysis of full discharges for supercapacitive MFCs

**Fig. 1:**
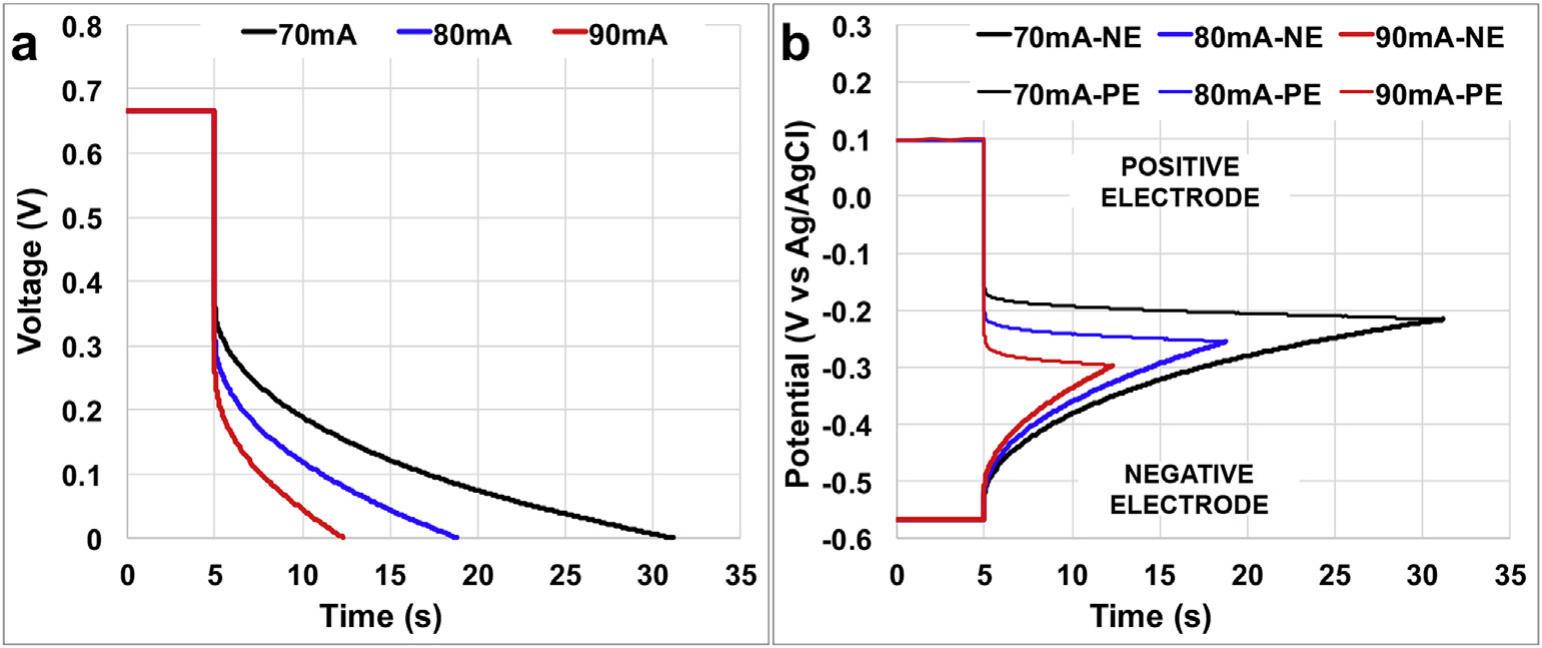
Full discharge cycles for the SC-MFC with electrolyte solution conductivity of 13 mS cm^−1^. Cell profile (a) and single electrode profiles (b).

Full discharge of the SC-MFC at i_pulse_ of 70 mA (70 A m^−3^), 80 mA (80 A m^−3^) and 90 mA (90 A m^−3^) are reported for the operating solution conductivity of 13 mS cm^−1^ (Fig. 1). SC-MFC had a **V**_**max,OC**_ of ≈667 mV that was due to the difference in potential between the positive electrode (≈+105 mV vs Ag/AgCl) and the negative electrode (≈-561 mV vs Ag/AgCl). The overall cell discharge, under this operating condition, showed a vertical drop of 326 mV (**i**_**pulse**_ 70 mA (80 A m^−3^)), 362 mV (i_pulse_ 80 mA (80 A m^−3^)) and 419 mV (**i**_**pulse**_ 90 mA (90 A m^−3^)) (Fig. 1a). These values showed that the SC-MFC **ESR** was 4.7 Ω. **R**_**NE**_ and **R**_**PE**_ were calculated from the single electrode profiles shown in Fig. 1.b and were 3.7 Ω and 0.9 Ω, respectively. This underlines that the majority of the ohmic losses were due to the positive electrode ohmic losses that counted as 84% of the total **ESR**. This result can also be explained by the presence of the ceramic separator between the electrolyte and the reference electrode that was certainly responsible for an increase in ohmic resistance and was previously described as **R**_**U**_. **C**_**stack**_ was evaluated for the longer discharge occurring at **i**_**pulse**_ of 70 mA (70 A m^−3^) in which the current was sustained for a duration of 26.2 s **ΔV**_**capacitive,stack**_ during the full discharge was 366 mV for **i**_**pulse**_ 70 mA (70 A m^−3^) (Fig. 1a) and therefore **C**_**stack**_ was quantified in 5.0 F. Single electrode profiles helped to calculate the contribution of each electrode that were 30.6 F and 6 F for **C**_**PE**_ and **C**_**NE**_ respectively. This result is quite expected due to the nature of the electrodes utilized during the experiments. The negative electrode in fact was based on carbon veil, which is a good electrode material as anode for MFC application but does not posses high surface area or supercapacitive features and therefore is not suitable for supercapacitor applications. On the contrary, high surface area activated carbon with Fe-based catalysts embedded into the mixture has high surface area possessing suitable characteristics for SC applications.

### 3.2 Analysis of SC-MFC for discharges at t pulse equal to 5s

**Fig. 2:**
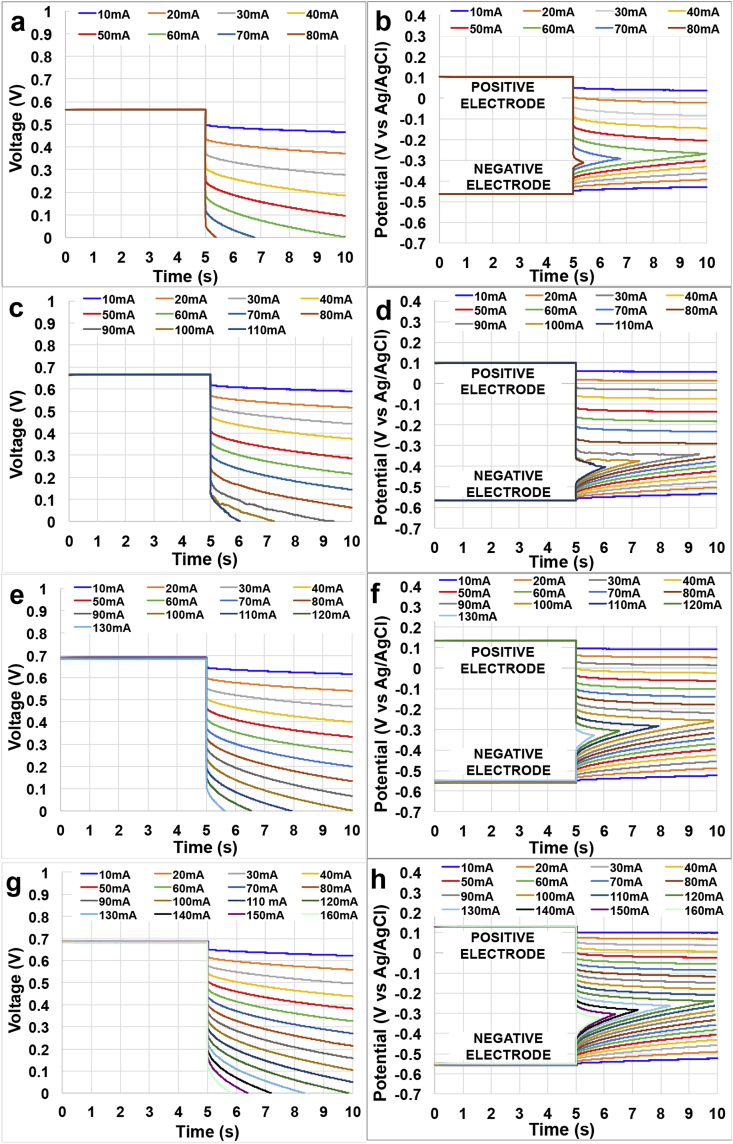
SC-MFC discharges at solution conductivity of 2.5 mS cm^−1^ (a), 13 mS cm^−1^ (c), 22 mS cm^−1^ (e) and 40 mS cm^−1^ (g). Single electrode profile during the discharges at solution conductivity of 2.5 mS cm^−1^ (b), 13 mS cm^−1^ (d), 22 mS cm^−1^ (f) and 40 mS cm^−1^ (h). All the discharges were done at a **t**_**pulse**_ of 5 s and at different **i**_**pulse**_.

Galvanostatic discharges of the ceramic MFC stack having different electrolyte solution conductivity for **t**_**pulse**_ of 5 s at different **i**_**pulse**_ are here reported (Fig. 2a, 2c, 2e and 2g). The profiles of the single electrode (positive and negative) during the discharges are also reported (Fig. 2b, 2d, 2f and 2h). Interestingly, **V**_**max,OC**_ increased moving from activated sludge (2.5 mS cm^−1^) to the other more conductive electrolytes investigated. In fact **V**_**max,OC**_ was roughly 570 mV at the lower ionic strength investigated with a contribution of ≈ -460 mV (vs Ag/AgCl) from the negative electrode and ≈+110 mV (vs Ag/AgCl) from the positive electrode (Fig. 2). At higher solution conductivity, **V**_**max,OC**_ increased to 675 ± 10 mV and this is due to the more negative potential at rest condition of the negative electrode (Fig. 2). In fact the contribution of the single electrodes are ≈ -551 ± 10 mV (vs Ag/AgCl) from the negative electrode and ≈+120 ± 10 mV (vs Ag/AgCl) from the positive electrode. This decrease in potential of the negative electrode might be due to the addition of acetate. The positive electrode potential is not affected significantly by the addition of acetate. The maximum current that can be achieved during GLV discharges enhanced with the increasing in solution conductivity indicating a positive effect of the latter on the performance (Fig. 2).

ESR was quantified in 6.0 Ω, 4.6 Ω, 4.3 Ω and 3.4 Ω for operating solution conductivities of 2.5 mS cm^−1^, 13 mS cm^−1^, 22 mS cm^−1^ and 40 mS cm^−1^, respectively. As can be seen in Fig. 2b, 2d, 2f and 2.h the contribution was greater from the positive electrode (**R**_**PE**_). In fact, **R**_**PE**_ was 4.7 Ω, 3.7 Ω, 3.3 Ω and 2.6 Ω respectively. **R**_**NE**_ was roughly constant and independent from the solution conductivity measuring 1.3 Ω, 0.7 Ω, 1.0 Ω and 0.8 Ω, respectively. **R**_**PE**_ decreased with the increasing of the solution conductivity probably for the reduction of the ohmic resistance within the electrolyte and the ceramic separator due to the enhancement in the ionic strength. Therefore **R**_**U**_ was significantly diminished. **R**_**PE**_ contributed by 77.5 ± 2.5% to the total **ESR**.

The apparent capacitive features of the stack (**C**_**stack**_) and of the single electrodes (**C**_**NE**_ and **C**_**PE**_) were evaluated for **i**_**pulse**_ of 60 mA. **C**_**stack**_ increased with the electrolyte solution conductivity measuring 1.5 F, 1.8 F, 1.9 F and 2.0 F for solution conductivity of 2.5 mS cm^−1^, 13 mS cm^−1^, 22 mS cm^−1^ and 40 mS cm^−1^, respectively. **C**_**NE**_ slightly decreased from 2.8 F to 2.5 F when the conductivity increased from 2.5 mS cm^−1^ to 13 mS cm^−1^ but then remained constant for the remaining electrolyte tested. **C**_**PE**_ instead increased significantly with the conductivity measuring 3.0 F, 6.4 F, 7.4 F and 9.6 F, respectively. The values measured here for the **C**_**PE**_ are similar compared to the one previously presented [78] but **C**_**NE**_ was roughly half, with a decrease of about 30% the overall **C**_**stack**_ compared to the previous work [78].

### 3.3 Power generated at different solution conductivities

**Fig. 3:**
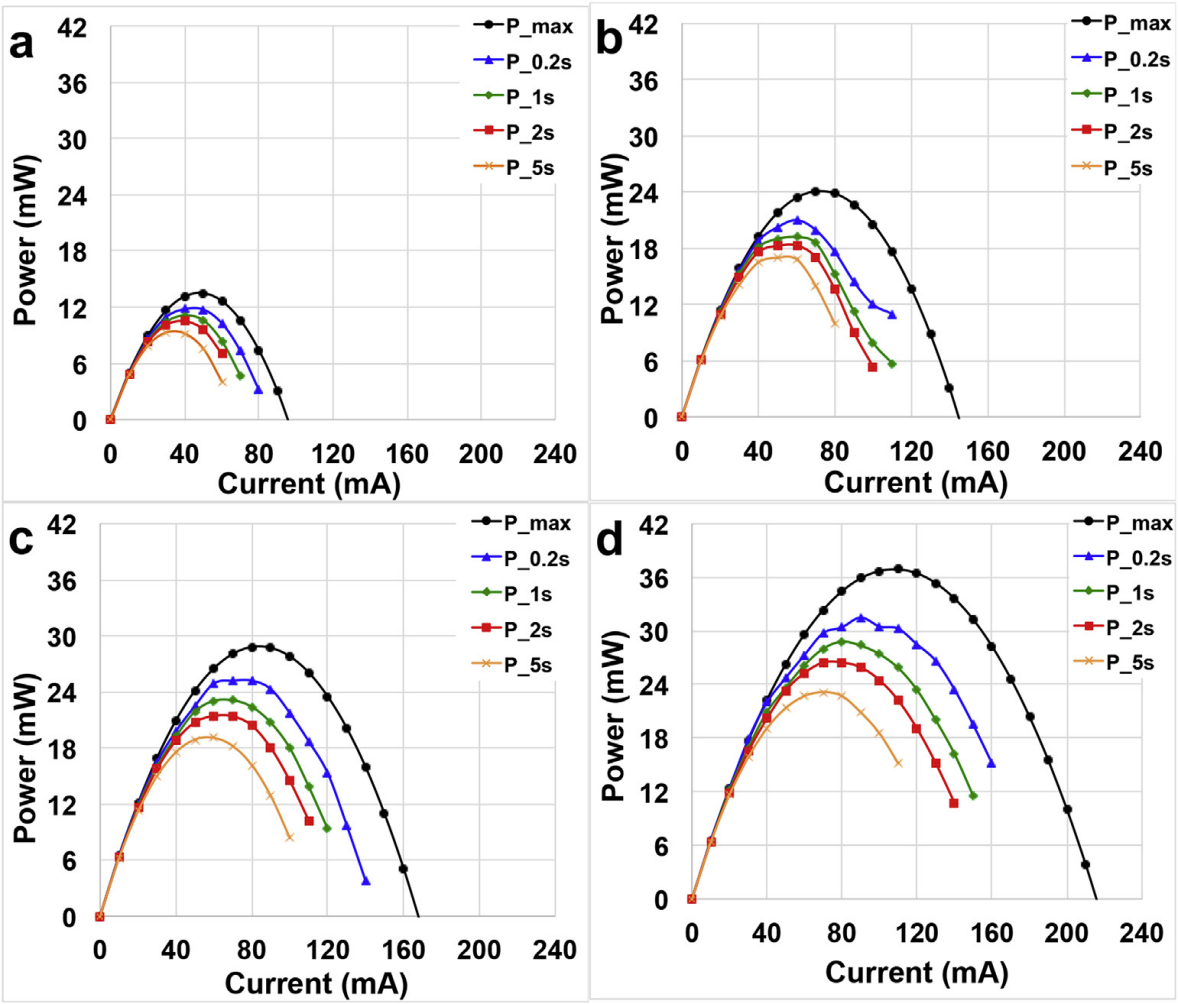
P_max_ curves and P_pulse_ curves with SC-MFC fed with electrolyte having solution conductivity of: (a) 2.5 mS cm^−1^, (b) 13 mS cm^−1^, (c) 22 mS cm^−1^, (d) 40 mS cm^−1^.

Power curves were calculated in terms of **P**_**max**_ and **P**_**pulse**_ for **t**_**pulse**_ of 0.2 s, 1 s, 2 s and 5 s. Power curves related to SC-MFC operating with activated sludge were reported in Fig. 3 a, SC-MFC operating at 13 mS cm^−1^ in Fig. 3.b, and with solution conductivity of 22 mS cm^−1^ and 40 mS cm^−1^ were reported in Fig. 3c and 3.d respectively. By definition, **P**_**max**_ is the maximum achievable power from the system if capacitive behavior was not present. **P**_**max**_ increased significantly with the solution conductivity measuring 13.5 mW (13.5 W m^−3^), 24.1 mW (24.1 W m^−3^), 28.8 mW (28.8 W m^−3^) and 36.9 mW (36.9 W m^−3^) at 2.5 mS cm^−1^, 13 mS cm^−1^, 22 mS cm^−1^ and 40 mS cm^−1^, respectively. **P**_**pulse**_ for **t**_**pulse**_ of 0.2 s, 1 s, 2 s and 5 s were lower than **P**_**max**_. **P**_**pulse**_ values decreased with the increase of the **t**_**pulse**_ since the capacitive behavior was considered. At solution conductivity of 2.5 mS cm^−1^, maximum **P**_**pulses**_ recorded were 11.8 mW (11.8 W m^−3^), 11.2 mW (11.2 W m^−3^), 10.5 mW (10.5 W m^−3^) and 9.3 mW (9.3 W m^−3^) for **t**_**pulse**_ of 0.2 s, 1 s, 2 s and 5 s. At solution conductivity of 13 mS cm^−1^, maximum **P**_**pulses**_ measured for **t**_**pulse**_ of 0.2 s, 1 s, 2 s and 5 s were 21.0 mW (21.0 W m^−3^), 19.2 mW (19.2 W m^−3^), 18.3 mW (18.3 W m^−3^) and 17.0 mW (17.0 W m^−3^), respectively. Maximum **P**_**pulses**_ increased more when SC-MFC was operated with solution conductivity of 22 mS cm^−1^. Particularly, the SC-MFC produced a **P**_**pulse**_ of 25.2 mW (25.2 W m^−3^), 23.1 mW (23.1 W m^−3^) and 21.4 mW (21.4 W m^−3^), 19.1 mW (19.1 W m^−3^) at **t**_**pulse**_ of 0.2 s, 1 s, 2 s and 5 s, respectively. Higher maximum **P**_**pulses**_ were achieved at higher solution conductivities. In fact, SC-MFC produced a **P**_**pulse**_ of 31.5 mW (31.5 W m^−3^), 28.8 mW (28.8 W m^−3^) and 26.5 mW (26.5 W m^−3^), 23.1 mW (23.1 W m^−3^) at **t**_**pulse**_ of 0.2 s, 1 s, 2 s and 5 s, respectively. **P**_**max**_ of 36.9 mW (36.9 W m^−3^) was the highest recorded in this work and was higher than 27.4 mW (27.4 Wm^-3^) recorded in a previous work with identical solution conductivity but AC-based cathodes were used as positive electrode [78]. The utilization of Fe-based catalysts enhanced the positive electrode potential and therefore the power/energy output [78]. In fact, **P**_**max**_ increased by 25% compared to the previous work.

### 3.4 Analysis of the 4500 cycles

**Fig. 4:**
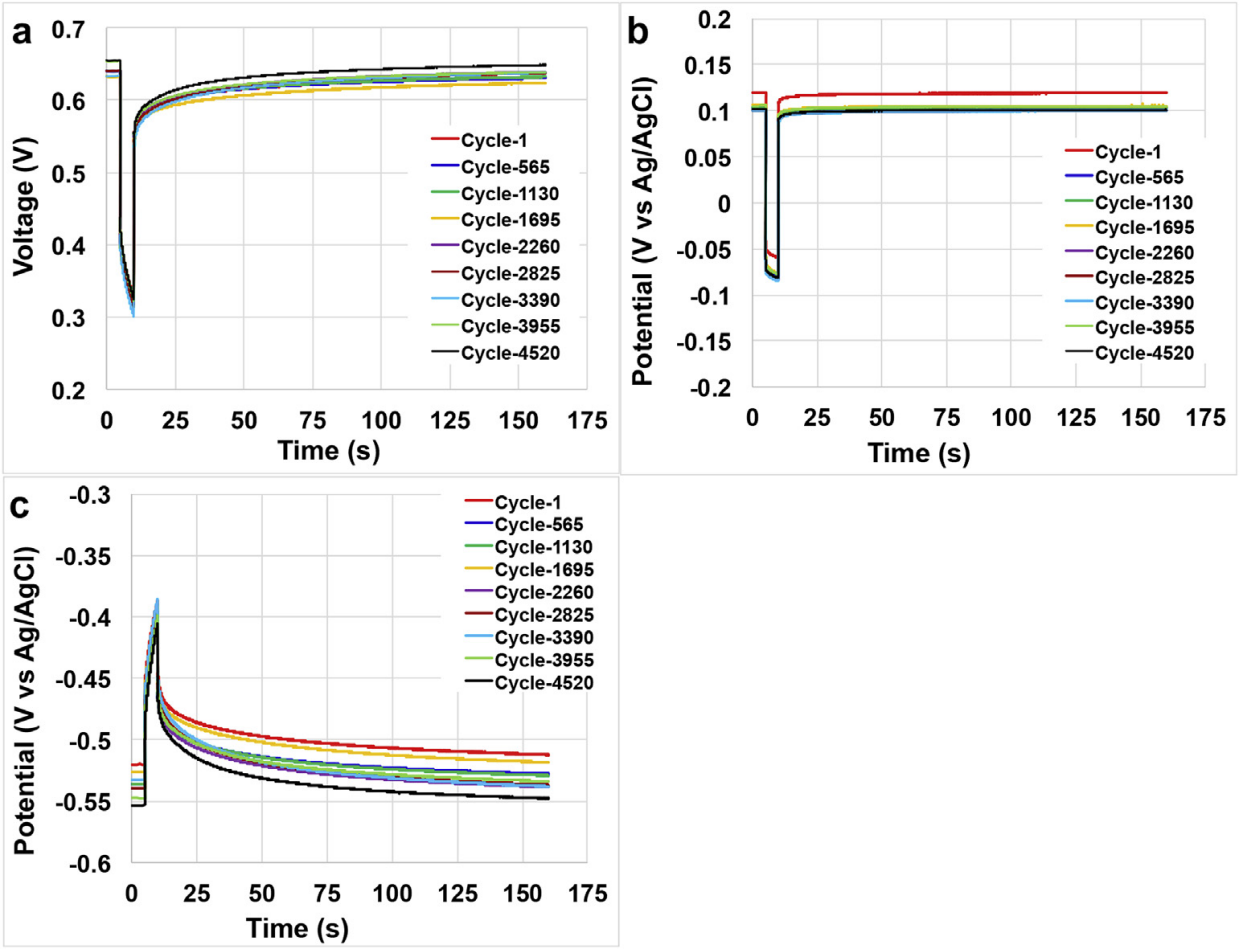
Discharge and self-recharge of the SC-MFC stack during the 4520 cycles operation. Stack voltage profile (a), positive electrode (b) and negative electrode (c) potentials trend.

Long term discharges and self-recharges were done on the SC-MFC with discharges of 55 mA (**i**_**pulse**_) for a t_pulse_ of 5 s and a self-recharge time (rest conditions) of 150 s in order restore the initial **V**_**max,OC**_. Discharges and self-recharges were run for a total of ≈8 days and the discharge/self-recharge profile of the overall SC-MFC stack (Fig. 4a), positive electrode (Fig. 4b) and negative electrode (Fig. 4c) of cycle 1 (day 0), cycle 565 (day 1), cycle 1130 (day 2), cycle 1695 (day 3), cycle 2260 (day 4), cycle 2825 (day 5), cycle 3390 (day 6), cycle 3955 (day 7) and cycle 4520 (day 8) are here presented. During this test, the solution was changed and fluxed within the SC-MFC. The solution used was prepared into a 4 L bottle and contained activated sludge with the addition of 500 mL of 100 g L^−1^ sodium acetate solution. The solution conductivity was 22.3 mS cm^−1^. This excess of carbon energy was intentional in order to provide enough organic compounds during the 8-day operations.

**Fig. 5:**
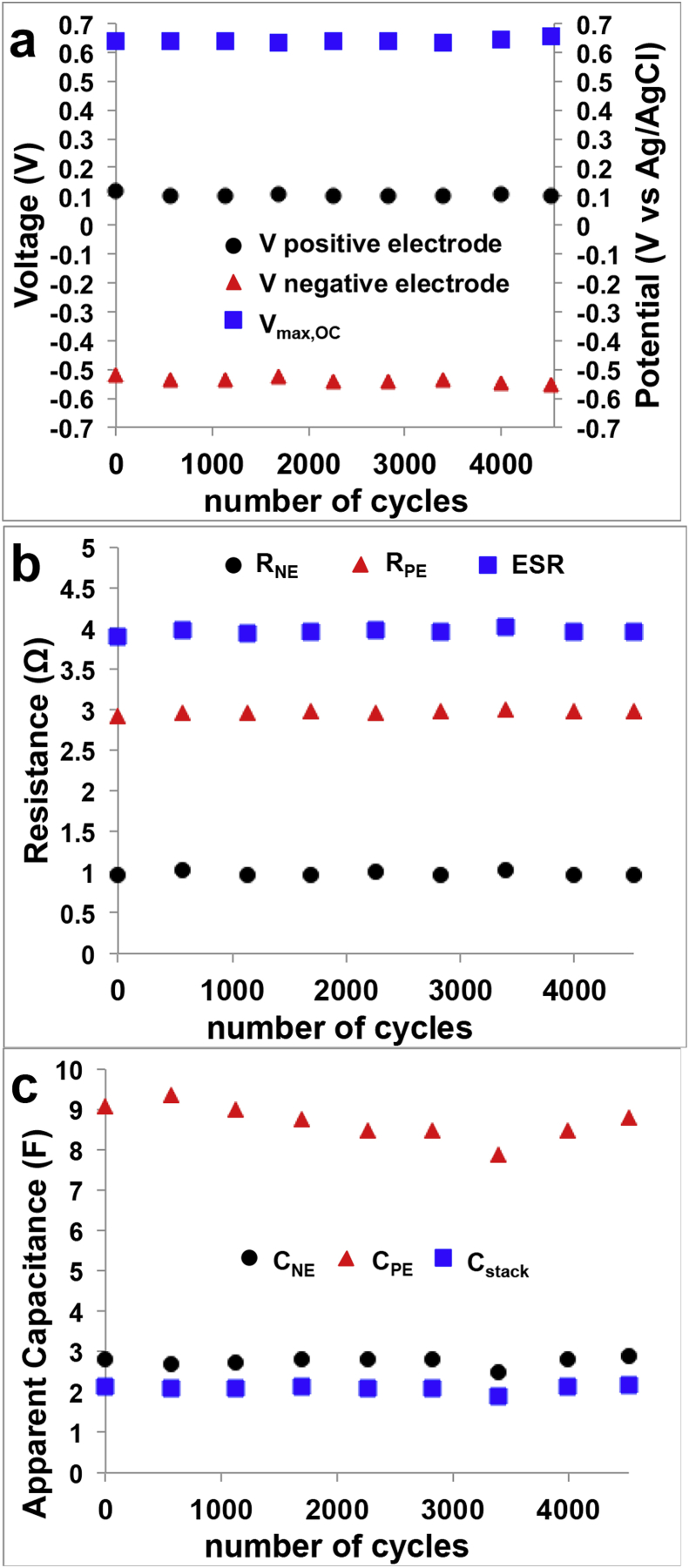
Trend over 4520 cycles of V_max,OC_ and positive/negative potential (a), R_NE_, R_PE_ and ESR (b) and C_NE_, C_PE_ and C_stack_.

As can be seen, a certain stability in the performance during the 8 days operations. The parameters of interest such as **V**_**max,OC**_, V negative electrode, V positive electrode (Fig. 5a), **ESR**, **R**_**NE**_, **R**_**PE**_ (Fig. 5b) and **C**_**stack**_, **C**_**PE**_, **C**_**NE**_ (Fig. 5c) were considered and described. The **V**_**max,OC**_ was stable over time with an average value of 640 ± 6 mV with the maximum achieved in the last cycle that was 654 mV. Considering the single electrode, the positive electrode had an average value of 104 ± 6 mV (vs Ag/AgCl) while the negative electrode measured −534 ± 10 mV (vs Ag/AgCl). It can be seen that the positive electrode slightly lowered its value moving from 120 mV (vs Ag/AgCl) at cycle 1–100 mV (vs Ag/AgCl) at cycle 4520. Similarly, also the negative electrode decreased from −520 mV (vs Ag/AgCl) at cycle 1 to −553 mV (vs Ag/AgCl) at cycle 4520. This might be due to the increase in the anaerobic conditions over time. Stability during long term operation was noticed also within the **ESR**, **R**_**PE**_ and **R**_**NE**_ with recorded values of 4.0 ± 0.1 Ω, 3.0 ± 0.1 Ω, 1.0 ± 0.1 Ω, respectively. Once again, the ohmic resistance due to the positive electrode (cathode and ceramic separator) counted for the 75% of the overall **ESR**. **C**_**stack**_ was stable over the 4520 discharges measuring 2.1 ± 0.1 F. **C**_**NE**_ and **C**_**PE**_ were 2.8 ± 0.1 F and 8.7 ± 0.4 F, respectively. In agreement with the discussion above, the lower apparent capacitance was due to the negative electrode affecting the overall SC-MFC apparent capacitance.

## 4 Outlook and conclusions

Ceramic MFC stack was used in supercapacitive mode considering the anode and the cathode of the MFC stack as the negative and positive electrode respectively of an internal supercapacitor. Intermittent MFC operations were shown to be beneficial in improving the power/current output [82,83]. Different solution conductivities were also investigated to simulate the output in the presence of different types of wastewater. Activated sludge itself was also used as the feeding source for the SC-MFC. Due to the high overall geometric electrode area utilized, which was 6720 cm^2^ for the negative electrode and 910 cm^2^ for the positive electrode, high current GLV discharges were performed. The increase in solution conductivity also enhanced the performance output lowering the positive electrode ohmic resistance and therefore the overall ESR. The positive electrode counted as 75–80% of the overall ESR and this high contribution was due to the presence of the ceramic separator that divided the positive electrode from the direct contact with the electrolyte separator. Despite the higher geometric area, the negative electrode had lower apparent capacitance compared to the positive electrode and this was probably due to the differences in the materials used for the electrodes. In fact, activated carbon has much higher specific surface area compared to carbon veil and therefore has better supercapacitive features and is more suitable for supercapacitive applications. The addition of a catalyst increased substantially the potential of the positive electrode to higher values giving at least an advantage of more than 50 mV compared to the values recorded in a previous report, adopting bare AC materials as the catalyst [78]. This increase in the positive electrode potential certainly enhanced the **V**_**max,OC**_ and therefore the overall output. The increase in power generation with the utilization of Fe-based catalyst was roughly 25% passing from 27.4 W (27.4 W m^−3^) of the previous work [78] to 36.9 mW (36.9 W m^−3^). For the first time, 8 days continuous operation of an SC-MFC was presented. The system showed relatively high stability over the time investigated and the 4520 cycles of discharge and self-recharge.

Despite relative high power generation is presented, several pathways need to be further investigated in order to further improve the system. Firstly, ESR still remained high and certainly hindered the overall power output therefore more conductive materials, connections and separators should be adopted. The increase in available **V**_**max,OC**_ is certainly a beneficial strategy as shown in this current investigation. The utilization of an additional electrode with supercapacitive features that overcome the ohmic losses of the limiting electrode could also be an option to be considered as shown before in SC-MFC [72]. Secondly, the materials utilized in this work as electrodes of the SC-MFC are not fabricated or considered for their supercapacitive features especially the carbon veil. Therefore apparent capacitance of the single electrode that influences the overall apparent capacitance of the system should be increased and optimized. These materials should not just increase the capacitive features of the electrode but should also warrant high **V**_**max,OC**_ and consequently high current/power pulses.
